# Development and Characterization of a Spontaneously Metastatic Patient-Derived Xenograft Model of Human Prostate Cancer

**DOI:** 10.1038/s41598-018-35695-8

**Published:** 2018-12-03

**Authors:** Tobias Lange, Su Jung Oh-Hohenhorst, Simon A. Joosse, Klaus Pantel, Oliver Hahn, Tobias Gosau, Sergey A. Dyshlovoy, Jasmin Wellbrock, Susanne Feldhaus, Hanna Maar, Renate Gehrcke, Martina Kluth, Ronald Simon, Thorsten Schlomm, Hartwig Huland, Udo Schumacher

**Affiliations:** 1 Institute of Anatomy and Experimental Morphology, University Medical Center Hamburg-Eppendorf, Martinistrasse 52, 20246 Hamburg, Germany; 2 Martini-Clinic, Prostate Cancer Center, University Medical Center Hamburg-Eppendorf, Hamburg, Germany; 3 Department of Tumor Biology, University Medical Center Hamburg-Eppendorf, Hamburg, Germany; 4 Department of Urology, University Medical Center Goettingen, Robert-Koch-Strasse 40, 37075 Goettingen, Germany; 5 Laboratory of Experimental Oncology, Department of Oncology, Hematology and Bone Marrow Transplantation with Section Pneumology, Hubertus Wald-Tumorzentrum, University Medical Center Hamburg-Eppendorf, Hamburg, Germany; 6 School of Natural Sciences, Far Eastern Federal University, Vladivostok, Russian Federation; 7 Department of Oncology, Hematology and Bone Marrow Transplantation with Section Pneumology, University Medical Center Hamburg-Eppendorf, Hamburg, Germany; 8 Institute of Pathology, University Medical Center Hamburg-Eppendorf, Hamburg, Germany; 9 Department of Urology, Charité University Hospital, Berlin, Germany

## Abstract

Here we describe the establishment and characterization of an AR+, PSMA+, ERG+, PTEN^−/−^, CHD1^+/−^ patient-derived xenograft (PDX) model termed ‘C5’, which has been developed from a 60 years old patient suffering from castration-resistant prostate cancer (CRPC). The patient underwent radical prostatectomy, showed early tumor marker PSA recurrence and, one year after surgery, abiraterone resistance. Subcutaneous C5 tumors can be serially transplanted between mice and grow within ~90 days to 1.5–2 cm³ tumors in SCID Balb/c mice (take rate 100%), NOD-*scid* IL2Rg^null^ (NSG) mice (100%) and C57BL/6 *pfp*^−/−^/*rag2*^−/−^ mice (66%). In contrast, no tumor growth is observed in female mice. C5 tumors can be cryopreserved and show the same growth characteristics *in vivo* afterwards. C5 tumor cells do not grow stably *in vitro*, neither under two- nor three-dimensional cell culture conditions. Upon serial transplantation, some C5 tumors spontaneously disseminated to distant sites with an observable trend towards higher metastatic cell loads in *scid* compared to NSG mice. Lung metastases could be verified by histology by means of anti-PSMA immunohistochemistry, exclusively demonstrating single disseminated tumor cells (DTCs) and micro-metastases. Upon surgical resection of the primary tumors, such pulmonary foci rarely grew out to multi-cellular metastatic colonies despite doubled overall survival span. In the brain and bone marrow, the metastatic cell load present at surgery even disappeared during the post-surgical period. We provide shallow whole genome sequencing and whole exome sequencing data of C5 tumors demonstrating the copy number aberration/ mutation status of this PCa model and proving genomic stability over several passages. Moreover, we analyzed genomic and transcriptomic alterations during metastatic progression achieved by serial transplantation. This study describes a novel PCa PDX model that enables future research on several aspects of metastatic PCa, particularly for the AR+ , ERG+ , PTEN^−/−^ PCa subtype.

## Introduction

Prostate cancer (PCa) is the most commonly diagnosed cancer in men in Western countries and the third leading cause of cancer-related deaths^1^. Based on autopsy studies, PCa has a prevalence of about 8%, of which only 50% are clinically apparent^2^. PCa has a very good prognosis with a relative five-year survival rate of 98% among all stages (SEER Cancer Statistics Review 1975–2014, National Cancer Institute). Patients with localized, low-risk PCa even show a survival benefit compared to the normal population^3^ so that active surveillance is an accepted treatment option. However, as soon as the tumor develops metastatic competence, the relative five-year survival rate drastically drops to about 30% (SEER Cancer Statistics Review 1975–2014, National Cancer Institute).

Over the past years, the treatment options for such advanced stage PCa patients were expanded by the discovery of different androgen deprivation therapy (ADT) regimens (*e.g*., bicalutamide, enzalutamide, abiraterone) as a ‘fourth pillar’ of PCa therapy besides conventional chemotherapy, surgery and radiotherapy. However, after initial remission upon ADT, many tumors return as castrate-resistant PCa (CRPC) that remain androgen-dependent, but are essentially untreatable and commonly develop distant metastases^4^. Another unresolved problem is that some PCa patients experience a prolonged period (>5 years) after surgical resection of the primary tumor with no evidence of disease before the disease recurs at a metastatic, aggressive stage^5^. This phenomenon can be explained by the common hypothesis that single disseminated tumor cells (DTCs) already exist at a distant site at the time of initial diagnosis and primary tumor resection and stay in a quiescent state of so-called metastatic dormancy. After highly variable periods of time, the DTCs overcome this stage and start to colonize the metastatic organ^6^. The mechanisms that regulate these processes of dormancy and reactivation, however, remain unclear^7^.

To address these key questions it is important to develop preclinical mouse models that reflect the pathophysiology of the human disease as closely as possible. Some research groups started to establish patient-derived xenograft (PDX) models, which are based on direct transplantation of cancer tissue samples from the patient onto immuno-deficient mice. Such models have meanwhile been established for a variety of human cancers and show promising results regarding the retention of the molecular and cellular characteristics of the primary patient tumor^8^. In PCa, both prostatectomy and biopsy specimens have already successfully been used for this purpose^9^, either as small tumor pieces or single-cell suspension, alone or in combination with Matrigel or mouse seminal vesicle mesenchyme (SVM) (recently reviewed in^10^). Moreover, different immuno-deficient mouse strains have already been tested such as nude (nu/nu) mice (lacking functional T cells), SCID and NOD-SCID mice (lacking functional T and B cells) or NOD-SCID/IL2γ-receptor null (NSG) mice (lacking functional T, B and NK cells), collectively indicating that more severely immuno-deficient mice are better suited for PDX generation^10^. In addition, different implantation sites have been compared such as the dorsal region (subcutaneous, s.c.), the subrenal capsule (subcapsular) and the anterior prostate (orthotopic). Tumor take rates in s.c. models are rather low ranging from 20–75% and are mostly successful when the donors suffer from advanced, metastatic tumors^11^ while the tumor take rate in subcapsular models ranges from about 50% to >90%^9,11,12^. Orthotopic implantation has an approximately 70% take rate, but is technically challenging^11^ and has been reported to develop lymph node, but not distant metastases^13^.

However, despite these advances, the majority of published PCa PDX models was particularly intended for the use in preclinical testing of the anti-tumor activity of novel drugs^10^. As outlined above, a suitable PDX model that enables studies on one of the least understood aspects of the metastatic cascade, *i.e*. the regulation of DTC dormancy or otherwise outgrowth and colonization, has to our knowledge not been published yet. Therefore, we aimed to establish a novel PDX model of human CRPC and to analyze potential metastatic progression during serial transplantation. The progression to a more malignant phenotype through serial transplantation has already been shown in ovarian cancer, where a mixed Müllerian tumor developed into a serous ovarian carcinoma during serial transplantation^14^. One particular focus was whether distant metastases were initially developed at the single DTCs level and whether such lesions grew out after prolonged periods. Moreover, the novel PDX model was aimed to be characterized concerning genomic and transcriptomic changes during metastatic progression.

## Materials and Methods

### Xeno-transplantation of prostatectomy specimen and serial transplantation

Approximately 0.5 g of the original human prostatectomy specimen was used for establishment of the PDX model (approved by the local ethics committee [Ethik-Kommission der Ärztekammer Hamburg] with the project number PV3552). All experiments were performed in accordance with all relevant guidelines and regulations and informed consent had been obtained from the donor. A small proportion of the tumor sample was fixed with 4% formalin for 24 h and embedded in paraffin for subsequent histology. The remaining tumor tissue was covered with cell culture medium RPMI-1640 (Gibco, Paisley, UK) and minced with a scalpel blade under sterile conditions. By using a syringe plunger, the minced tissue was ground through a 100 µm mesh size cell strainer (Corning, VWR, Darmstadt, Germany), which was placed into a 50 ml Falcon tube. The cell strainer was rinsed with medium and the resulting tumor cell suspension in the Falcon tube was washed once. The cell pellet was resuspended with cooled medium mixed 1:2 with growth factor-reduced Matrigel (BD, Franklin Lakes, USA) in a total volume of 200 µl. This suspension was injected subcutaneously (s.c.) into the subscapular region of a male, 12 weeks old SCID Balb/c mouse (Charles River, Sulzfeld, Germany).

When s.c. xenograft tumors reached ~1.5 cm³, they were serially transplanted onto new recipients. For this purpose, tumor-bearing mice were anesthetized by intraperitoneal injection of ketamine hydrochloride (100 mg/mL, 1.2 mL/kg; Graub, Bern, Switzerland) and xylacine hydrochloride (20 mg/mL; 0.8 mL/kg; Bayer, Leverkusen, Germany). Next, 200 µl of EDTA blood were taken by cardiocentesis (and subjected to DNA isolation) and mice were sacrificed by cervical dislocation. Afterwards, the left lung as well as parts of the brain and liver were resected and freshly frozen for subsequent DNA isolation. The right lungs were prepared for histology as previously described^15^. The bone marrow was flushed out of the left femur and tibia using 0.9% NaCl (700 µl each, collected in an Eppendorf tube) and subjected to DNA isolation. Finally, the PDX tumors were harvested and one half was prepared for re-injection as described above. The rest of the tumor was cut into several pieces for formalin-fixation (histology), DNA- (fresh frozen) or RNA-isolation (frozen in 500 µl QIAzol Lysis Reagent, Qiagen), as well as *in vitro* cultivation (see below).

### Use of different immunodeficient mouse strains, female mice and transient cryopreservation

After the first 6 passages in SCID mice, the growth of C5 tumors was tested in alternative immunodeficient mouse strains, namely NOD-*scid* IL2Rg^null^ (NSG) mice (The Jackson Laboratory, Bar Habor, USA) and C57BL/6 *pfp*^−/−^/*rag2*^−/−^ mice (Taconic, Hudson, USA). In addition, after passage 8, the C5 tumors of three male NSG donors were transplanted onto one age-matched male and one female NSG mouse each to determine gender differences in the tumor take rates. Furthermore, beginning at passage 6, one half of the harvested tumors was minced with the scalpel blade and ground through the cell strainer, but prepared for transient cryopreservation after washing. For this purpose, the washed pellet was resuspended with CryoSafe Medium (cryo-safe I KM-11-D, c.c.pro, Oberdorla, Germany) and stored at −80 °C. Several of these cryopreserved C5 tumor aliquots were checked for *in vivo* tumor growth after thawing using SCID and NSG mice.

### Surgical resection of primary tumors

As a further modification of the protocol, beginning after passage 6, additional NSG mice were engrafted with C5 tumors and tumors were surgically resected when reached about 1.5 cm³. Briefly, mice anesthetized with ketamine/xylazin were depilated in the tumor area and placed on a warming mat. The skin above the tumor was incised, the tumor was mobilized and cut off the underlying connective tissue and supplying microvessels using an electrocautery device. Skin closure was performed with disposable skin staples (3 M Health Care, Borken, Germany) and mice received Carprofen s.c. (5 mg/mL, 5 mg/kg, Zoetis, Berlin, Germany) immediately after surgery as well as on the first and second post-operative day. Skin staples were removed on the seventh post-operative day. Mice were monitored after surgery for at least the primary tumor growth span before they were sacrificed and distant organs harvested for detection of metastatic cells as described above. All animal experiments described in this study were approved by the local animal experiment approval committee (Behörde für Gesundheit und Verbraucherschutz, Amt für Verbraucherschutz, Lebensmittelsicherheit und Veterinärwesen, assigned project No. 88/09, 19/15 and 55/16), supervised by the institutional animal welfare officer and performed in accordance with relevant guidelines and regulations.

### Alu-PCR and histology for quantification of metastatic cell loads in the blood and distant organs

DNA isolation and *Alu*-PCR-based quantification of metastatic cell loads in the blood, lung, liver, brain and bone marrow have been performed as previously described^16^. Representative lung sections for immunostainings and standard H&E were generated as described elsewhere^15^.

### Immunohistochemistry

Immunohistochemical detection of AR and ERG was performed as previously described^17^. For detection of prostate-specific membrane antigen (PSMA), xenograft primary tumor and corresponding lung tissue sections were de-paraffinized and pre-treated with Dako Retrieval solution S2367 (pH 9, Dako, Hamburg, Germany) for 2 × 4 min in a microwave. The primary antibody against human PSMA (Dako M3620) was diluted 1:111 in Dako antibody diluent (final concentration 163.8 µg/mL) and incubated on the slides for 60 min at room temperature. For antibody detection, the Dako REAL^TM^ Detection System (K5005) was used according to the manufacturer’s instructions. Nuclei were counterstained using Mayer’s hemalum solution.

### Fluorescence *in-situ* hybridization

Four micrometer paraffin-embedded xenograft tumor sections were used for fluorescence *in-situ* hybridization (FISH). Tumor sections were incubated for 16 h at 50 °C, de-waxed, air-dried, and dehydrated in 70%, 85%, and 100% ethanol. Slides were pretreated in VP 2000 Pretreatment Reagent (Abbott, Des Plaines, USA) for 15 min at 80 °C, followed by 150 min incubation at 37 °C in 0.5% protease 1 solution (Abbott, Des Plaines, USA). 4 µl of FISH probe mix in 70% formamide 2x SSC solution was applied to the slides and co-denatured with the cellular DNA in a Hybrite hybridization oven for 10 min at 72 °C prior to overnight-hybridization at 37 °C in a humidified chamber. Two FISH probes were used: first, a spectrum-orange labeled CHD1 probe (made from BAC RP11–533M23 and RP11–422M08), and a spectrum-green labeled, commercial centromere 10 probe (#06J36–090; Abbott, Wiesbaden, Germany) as a reference; second, a spectrum-orange labeled PTEN probe (made from BAC RP11–380G5 and RP11–813O3), and a spectrum-green labeled, commercial centromere 10 probe (#06J36–090; Abbott, Wiesbaden, Germany). After hybridization, slides were subjected to serial stringent washings (2x SSC solution with 0,3% NP40 at 72 °C for 2 minutes) and counterstained with 0.2 µmol/L 4′-6-diamidino-2-phenylindole (DAPI) in antifade solution. Stained slides were manually interpreted under an epifluorescence microscope, and the predominant green and orange FISH signal numbers were recorded. Homozygous deletion was defined as complete lack of FISH signals in the cell nuclei, but presence of centromere 10 signals. Normal copy number status was defined as the presence of equal numbers of gene (PTEN/CHD1) signals and centromere 10 signals.

### Next Generation Sequencing

Tumor DNA was isolated using a Qiagen DNA Mini Kit. Isolated DNA underwent whole exome sequencing using the Ilumina HiSeq. 4000 platform. Data analysis were performed using our custom NGS pipeline as described elsewhere^18^, using Control-FREEC to evaluate the copy number aberrations^19^ and ANNOVAR to annotate variants present in the COSMIC database v85^20^.

### RNA sequencing

RNA was isolated from frozen tumor/QIAzol samples after mechanical homogenization in liquid nitrogen using a mortar. For RNA isolation, a standard phenol-chloroform extraction protocol was used. RNA integrity was checked using Bioanalyzer 2100 (Agilent Technologies). Library preparation was done with a total amount of 1 µg RNA per sample using the TruSeq RNA Library Prep Kit v2 (Illumina Inc., San Diego, CA, USA). Size range was checked to be around 280 bp using BioAnalyzer. Sequencing was done at the Transcriptome and Genome Analysis Laboratory (TAL) at the University Medical Center Goettingen on an Illumina HiSeq. 4000 platform. Results were processed using the BaseCaller to bcl files function in the Illumina software. De-multiplexing was done using bcl2fastq (version 2.17.1.14). Fastq files were then mapped to the human transcriptome (UCSC HG19) and the murine transcriptome (UCSC MM9) using TopHat2 (version 2.1.0)^21^ with very sensitive Bowtie2 settings. The Cuffdiff function of the Cufflinks package (version 2.2.1) was used to analyze differential gene expression^22^.

## Results

### Patient history of the C5 donor

A spontaneously metastatic PCa PDX model termed ‘C5’, which was obtained from a 60 years old high risk PCa patient during radical prostatectomy at the Martini-Clinic (Hamburg-Eppendorf) in March 2014, was established by primary transplantation onto a male, 12 weeks old SCID Balb/c mouse. The patient was firstly diagnosed with PCa in November 2013 with an initial Gleason of 5 + 4 in 12 of 12 cores and a PSA of 95 ng/mL. Although the initial staging by bone scan and computer tomography (CT) provided no clear evidence of distant metastasis, a neoadjuvant hormone therapy with buserelin s.c. (GnRH) for 3 months was initiated, due to the very high risk constellation as well as highly suspect digital rectal examination (DRE). Subsequent re-staging by magnetic resonance imaging (MRI) demonstrated a moderately downsized, locally advanced prostate cancer with suspicion of seminal vesicle and bladder neck infiltration which appeared to be resectable. At this stage, there was still no sign of any distant metastasis, even though the PSA level was only moderately reduced. Consequently, the patient underwent radical prostatectomy in March 2014, when the PDX material for the C5 model was obtained. The final histopathologic assessment of the specimen revealed Gleason 5 + 4, pT3b, pN1 (2/12), R1. The PSA level at surgery was 19 ng/mL. Based on the final histology, the decision to continue ADT in adjuvant intention has been made. The first postoperative PSA level 6 weeks after radical prostatectomy was 1.38 ng/mL. Within few months after surgery, PSA levels were rapidly increasing to 400 ng/mL despite adjuvant ADT, so that re-staging was initiated. Here, newly developed lymph node and bone metastases were observed explaining the increasing PSA levels under ADT. Because of the metastatic CRPC features, abirateron acetat (Zytiga®) was applied since June 2015. Unfortunately, the patient did not respond to the novel antiandrogen and the PSA values further increased to up to 800 ng/mL within 2 months under Zytiga. Therefore, the patient was treated with 12 cycles of docetaxel and regular denosumab injections in palliative intention until August 2016, when he died. To summarize, the clinical features of the C5 donor can be characterized by the limited response to current ADT, rapid disease progression by metastasis, and primary resistance to abiraterone.

### Tumor growth and metastatic spread during serial transplantation

**Figure 1:**
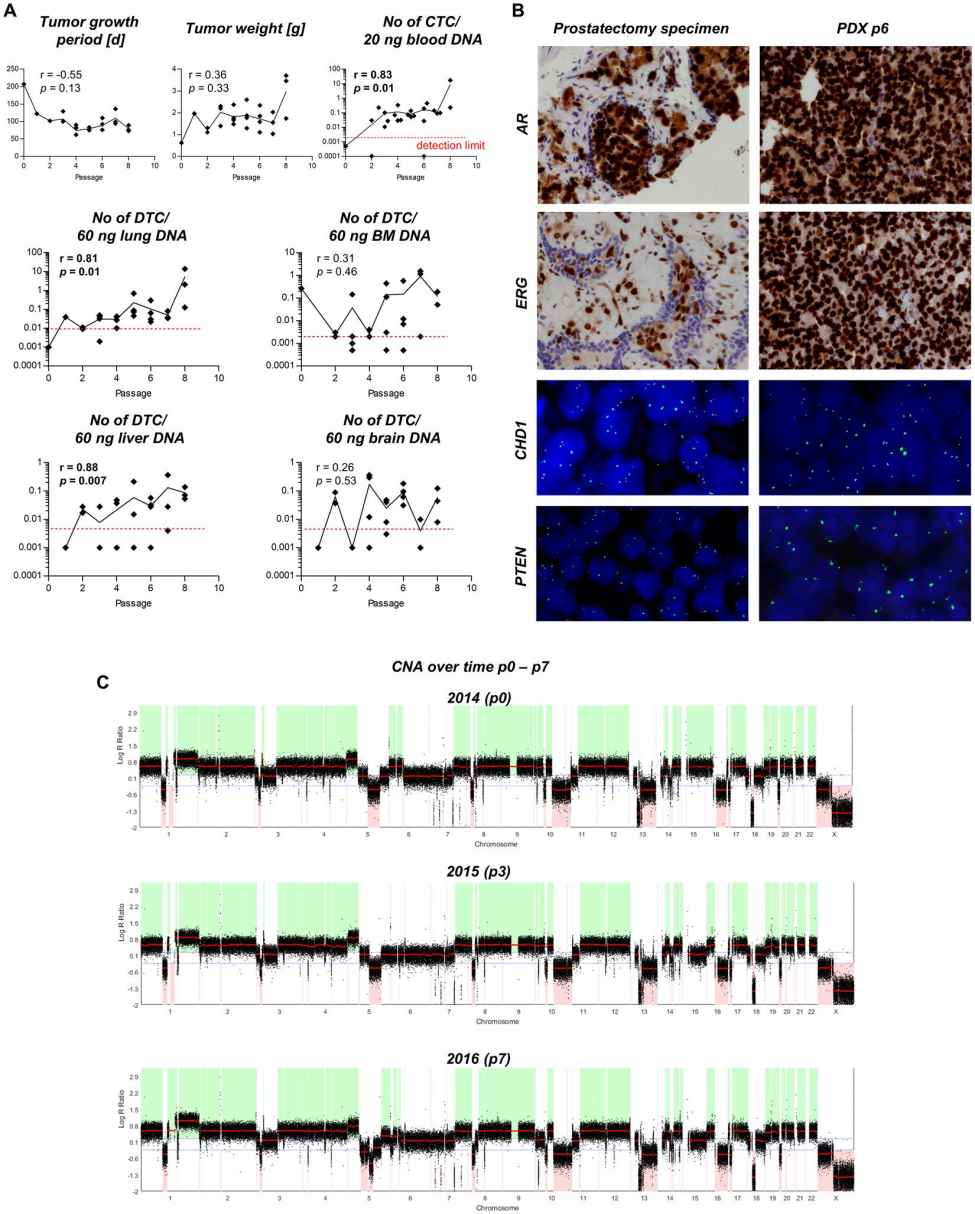
Characterization of the patient-derived xenograft (PDX) prostate cancer (PCa) model ‘C5’. (**A**) Subcutaneous tumor growth and spontaneous metastasis patterns to different distant sites among serial transplantation (passage 0 to 8) in 12-weeks old, male SCID mice. r- and *p*-values were calculated by Spearman correlation (significant correlations displayed in bold type). The red dotted lines indicate the detection limit for specific human sequences as determined by *Alu*-PCR. The black lines connect the mean values of each passage. (**B**) Androgen receptor (AR) and ETS-related gene (ERG) expression (IHC) as well as chromodomain helicase DNA binding protein 1 (CHD1) and phosphatase and tensin homolog (PTEN) deletion status (FISH) in original patient and PDX tumor at passage 6. (**C**) Copy number aberrations (CNA) determined by shallow whole genome sequencing at different passages (p) as indicated. CTCs = circulating tumor cells; DTCs = disseminated tumor cells; BM = bone marrow.

The initial C5 PDX tumor (passage 0) grew within 207 days and the resulting s.c. primary tumor weight was 0.62 g. The only distant site containing human DNA at passage 0 was the bone marrow (*Alu* qPCR signal >2 log10 levels above detection limit) with a DNA content of 0.26 human cells per 60 ng total DNA (~0.26 human cells per 10,000 host cells) at necropsy (Fig. 1A). In the following passages, the tumor growth periods were much shorter and ranged from 62 to 122 days. However, there was no significant inverse correlation between tumor growth period and number of passages. Likewise, the resulting s.c. tumor weights did not correlate with the number of passages. In contrast, the number of human cells in the blood, lung and liver correlated directly with the number of serial transplantations indicating metastatic progression over time. Such correlation could not be observed for the bone marrow or brain (numbers of human cells per DNA template for each distant site and passage as well as site-specific detection limits and results of Spearman correlations are shown in Fig. 1A).

C5 tumors maintained the key characteristics of the original tumor for at least 6 passages and are AR+, PSMA+, ERG+, PTEN^−/−^, CHD1^+/−^ (Fig. 1B). WB and qPCR revealed very low expression levels of AR-V7 at several passages (Suppl. Fig. S2). The only AR gene mutation detected by WES is a silent mutation (Chr X position 67545785 G→A; aa sequence: E→E). The C5 tumor cells are mainly triploid. Shallow whole genome sequencing (SWGS) was used to determine the molecular subtype of the C5 tumors and to investigate genomic stability from p0 to p3 and p7. By this we revealed a comparatively high number of heterozygous deletions (*e.g*., on the p arm of chromosomes 3, 8, 10, 17, 20 and on the q arm of chromosomes 5, 10, 13, 16) and a few homozygous deletions such as 7q, 13q 18q, Xq. Only very few differences between the different passages in copy number aberrations (CNA) could be observed, including on chromosome 5q, 6p and 15q (Fig. 1C). Supplementary Table T1 provides all CNA determined among these passages.

### Tumor growth on different immunodeficient backgrounds, after cryopreservation and on female mice

**Figure 2:**
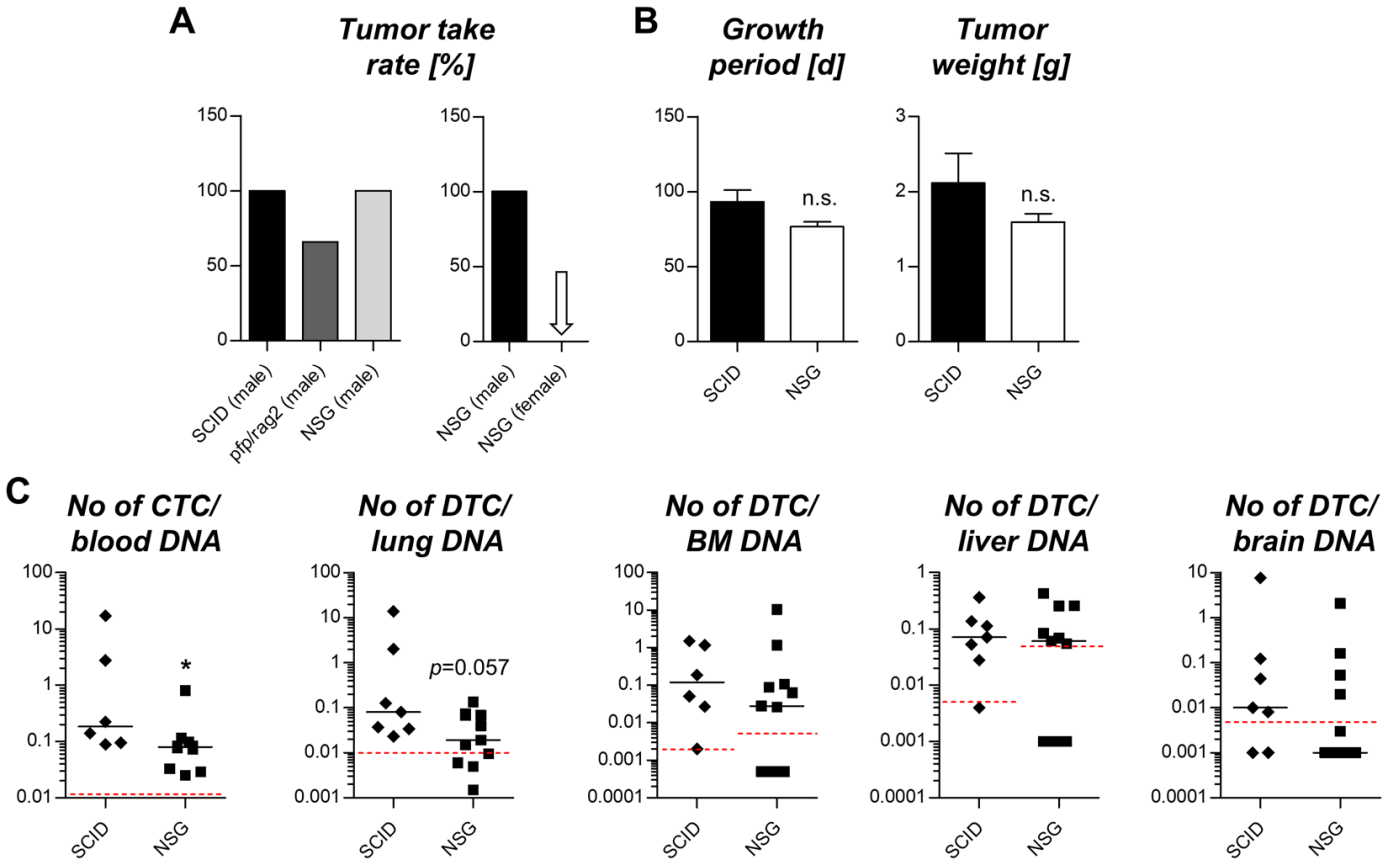
Comparison of C5 tumor growth and metastasis in different immunodeficient mouse strains. (**A**) Tumor take rates in male SCID (Balb/c background), NSG (NOD background) and *pfp*^−/−^/*rag2*^−/−^ (C57BL/6 background) mice. C5 tumors do not grow in female NSG mice (arrow). (**B**) Growth periods and resulting tumor weights at necropsy in SCID *vs*. NSG mice. (**C**) Metastatic cell loads at necropsy at different distant sites in SCID *vs*. NSG mice. The red dotted lines represent the detection limit for specific human sequences as determined by *Alu*-PCR for each immunodeficient background. Bar charts in (**B**) represent mean + standard deviation of n = 7 (SCID) and n = 10 (NSG); *p < 0.05 (Mann-Whitney test); n.s. = not significant. SCID = severe combined immunodeficiency; NSG = NOD-*scid* IL2Rg^null^; NOD = non-obese diabetic; CTCs = circulating tumor cells; DTCs = disseminated tumor cells.

There was no difference in the tumor growth characteristics in SCID (Balb/c background) *vs*. NSG (NOD background) mice (Fig. 2A,B) while *pfp*^−/−^/*rag2*^−/−^ mice (C57BL/6 background) showed tumor growth in only 66% of the tested mice (Fig. 2A). Therefore, metastasis patterns were compared in more detail in SCID *vs*. NSG mice demonstrating significantly more human cells in the blood and nearly significantly more human cells in the lung of SCID mice whereas other distant sites showed no difference in the number of human cells between SCID and NSG mice (Fig. 2C).

After transient cryopreservation, thawing, and re-injection in Matrigel, C5 tumors developed normally in terms of growth periods and tumor weights with a >90% tumor take rate (data not shown). To assess androgen-dependent growth of C5 tumors, tumor samples from three different male NSG donors were transplanted onto one male and one female NSG mouse each (age-matched). All male mice carried ~1.5 g tumors within 80 days while not any of the female mice showed tumor growth, not even after 160 days (Fig. 2A).

### Histologic verification of lung metastases and genomic/transcriptomic profiling

**Figure 3:**
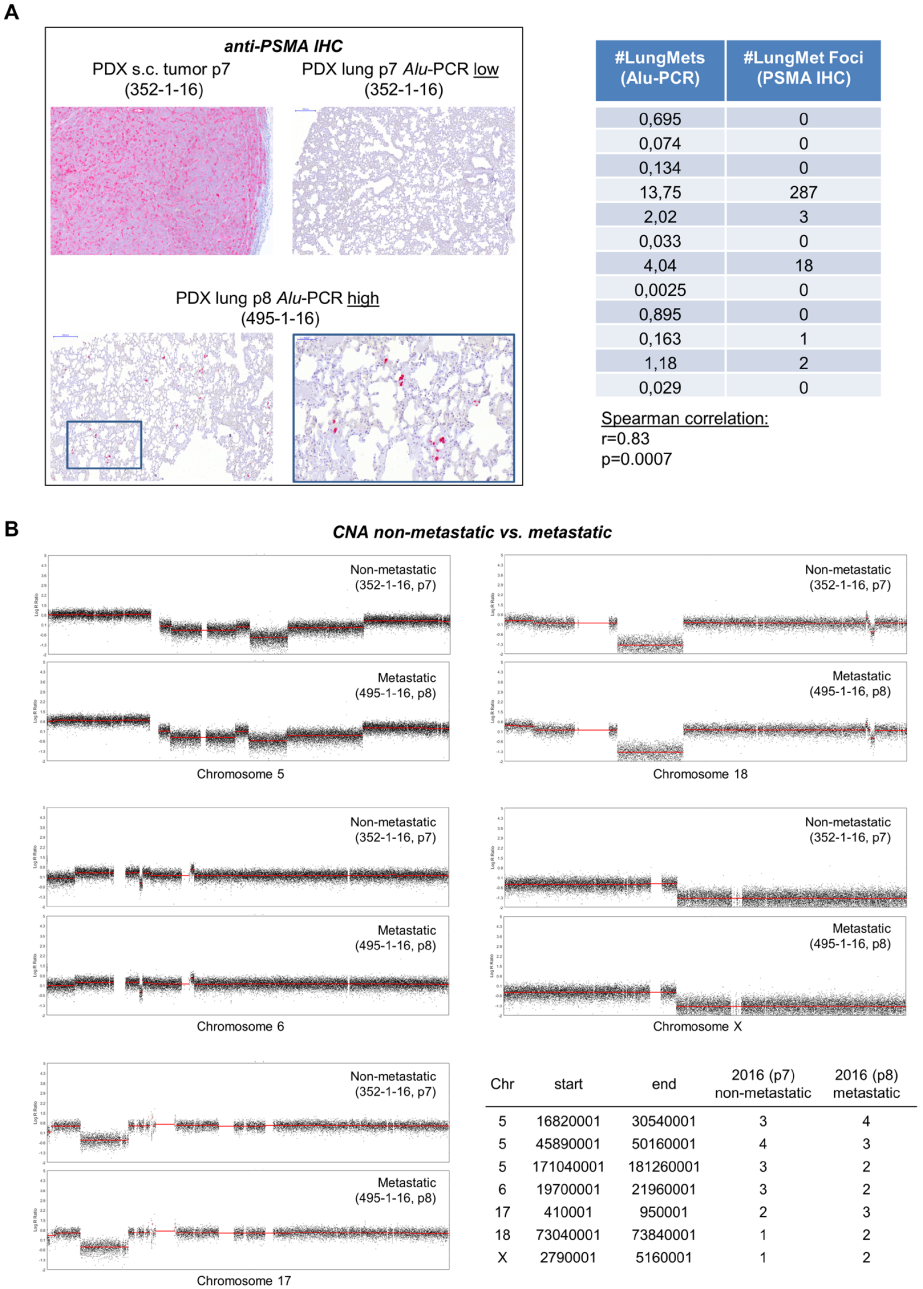
Prostate-specific membrane antigen (PSMA) expression and copy number aberrations (CNA) in non-metastatic *vs*. metastatic C5 xenografts of consecutive passage. (**A**) PSMA expression in primary C5 tumor and corresponding lung of a non-metastatic ‘parental’ mouse (passage7, #352–1–16) showing no PSMA staining in the lung. The contralateral lung contained very few human cells as per *Alu*-PCR (*Alu*-PCR low: 0.03 cells per 10,000 host cells). The lung of the ‘filial’ mouse (passage 8, #495-1-16) shows multiple PSMA-reactive DTCs. The contralateral lung contained a comparatively high number of human cells as per *Alu*-PCR (*Alu*-PCR high: 13.7 cells per 10,000 host cells). The embedded table provides the raw data of the 12 mice with the highest human cell load in the lung as per Alu-PCR from the total cohort, which were histologically examined using anti-PSMA IHC. Note the high Spearman correlation of r = 0.83 and significant p-value indicating close correlation between Alu-PCR- and PSMA-IHC-based quantification of lung metastases. (**B**) Shallow whole genome sequencing of C5 tumors of these two passages reveals few, insignificant alterations in the CNA on chromosomes 5, 6, 17, 18, and X as indicated. For transcriptomic profiling of non-metastatic *vs*. metastatic C5 pairs, please see Suppl. Tables T2 + 3 (human) and T4 + 5 (murine).

To validate the *Alu*-PCR results, 10 representative, H&E-stained lung tissue sections have been histologically examined per mouse as described before^15^. However, based on H&E histology, human tumor cells could not be detected. Therefore, C5 primary xenograft tumors were tested for expression of prostate-specific membrane antigen (PSMA). As primary tumors showed homogenous, moderate PSMA expression (Fig. 3A), the immunostaining for PSMA was used to detect metastatic deposits in the lungs. By this approach, we exclusively determined single DTCs and micro-metastases (2–3 cells) in the left lungs of mice whose right lungs contained relatively high numbers of human cells as determined by *Alu*-PCR (see table embedded in Fig. 3A). Lungs from mice with lower cell loads were entirely PSMA-negative (Fig. 3A). Lungs from healthy control mice did not show any PSMA-stained cells. Isotype controls of the used primary antibody did not reveal any staining on lung sections from mice with high *Alu*-PCR result (data not shown). Hence, both techniques used for detection of metastatic foci (*Alu*-PCR and PSMA IHC) correlated well (r = 0.83, p = 0.0007, Spearman correlation, Fig. 3A). The majority of analyzed mice revealed *Alu*-PCR signals above the detection limit of 0.01 human cells per 10,000 host cells, but showed no detectable anti-PSMA-reactive foci in the contralateral lung suggesting the presence of a few single DTCs in the majority of the mice.

Based on the finding of metastatic progression upon serial transplantation (see above, Fig. 1A) we next selected a C5 tumor pair from consecutive passages showing relatively strong differences in the metastatic cell loads at the different distant sites. From this metastatic parental *vs*. metastatic filial tumor, we isolated DNA from the bulk tumors and performed SWGS and WES. Interestingly, we did not find significant chromosomal gains or losses among metastatic progression; all observed slight alterations are included in the table embedded in Fig. 3B.

Two additional pairs of non-metastatic parental *vs*. metastatic filial C5 tumors were subjected to RNA-sequencing. Significant regulation was assigned when the FDR was below 0.05. Only one of these two pairs showed significant regulation of a few human genes (11 up-, 9 down-regulated in the metastatic filial tumor, Suppl. Table T2 + 3) while both analyzed pairs showed regulation of a total number of 38 murine genes (28 up-, 10 down-regulated in the metastatic filial tumor, Suppl. Table T4 + 5). All human and murine candidates (and those with q > 0.05, but p < 0.01) identified in both pairs are uploaded as Supplementary Tables 2–5 There was no overlap of murine genes regulated in both analyzed pairs.

### Metastatic outgrowth after primary tumor resection

**Figure 4:**
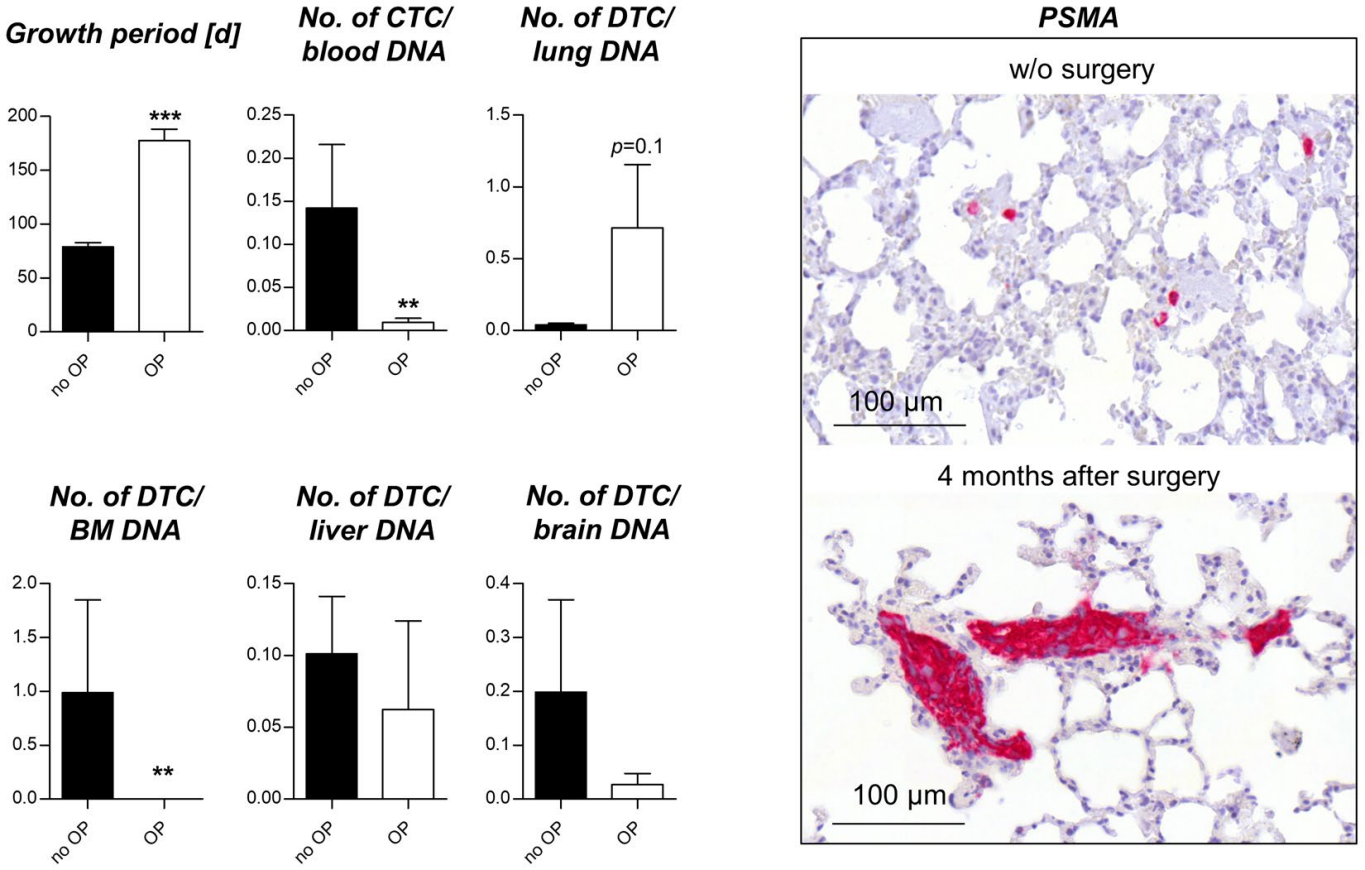
Persistence of DTCs at several sites after primary tumor surgery. C5 primary tumor resection (OP) prolonged the average growth period by ~100 days compared to untreated controls (no OP, end point: s.c. C5 tumor size of 1.5 cm³). Note the sharp decrease in CTCs and DTCs in the BM after this post-surgical period. At other sites, metastatic cell loads remain stable or even show a trend towards higher levels after surgery (lung). This tendency is due to very few individual mice showing multicellular colonies in the lungs as illustrated by PSMA immunostaining (2 of 10 mice).

The next question was whether the DTCs and micro-metastases grow out to multicellular colonies after prolonged growth periods. To address this question, we surgically resected the s.c. primary tumors to prolong the overall survival of the mice. The mean primary tumor weight at surgery was 1.72 g and the mean survival increased from 78.9 days to 177.5 days by surgery. After this post-operative period of average ~100 days, the number of CTCs in the blood and DTCs in the bone marrow was drastically reduced (*p* < 0.001, Mann-Whitney test). The metastatic cell loads at other sites, however, were unchanged or even showed an observable trend towards higher levels specifically in the lung. This trend was due to a few individual mice (2 of 10 surgically treated mice) showing multicellular lung colonies after the post-surgical monitoring period as validated by PSMA immunostaining (Fig. 4). However, the majority of mice did not show any significant alteration of the pulmonary or hepatic metastatic cell load after surgery.

## Discussion

Most of the published PDX studies were not intended to investigate the metastatic behavior of PCa^10^, and, if so, compared the metastatic potential of PDX tumors derived from different primary tumor foci of the same donor^23^. In sharp contrast, we analyzed metastatic progression during serial transplantation of a PDX model derived from one focus of one donor. Interestingly, the bone marrow was the first distant site that contained human cells already at passage 0 and showed no increase in the number of DTCs over time as observed for the blood, lung and liver. In the view of our data, the bone marrow is not more strongly affected by DTCs as a result from the metastatic progression achieved by serial transplantation while other organs become increasingly infiltrated (lung and liver). This would be in line with previous reports describing the bone marrow as a ‘sanctuary site’ of DTCs in terms of being an early and transient niche for metastasizing tumor cells^24^. This transient character of the bone marrow niche is further underlined by the sharp decrease in DTC numbers after surgical resection of the primary tumor in our study. In the view of these findings it might be that the metastatic cells in the bone marrow have to be constantly replaced by cells newly delivered from the primary tumor. In contrast, other distant sites such as lung and liver promote the survival of DTCs independently of the primary tumor (for at least 100 days) whereas metastatic outgrowth of these DTCs to multicellular colonies could be observed in a few individual mice only (in the lung). Therefore, we conclude that the majority of DTCs already present in the lung and liver during surgery remained in a state of metastatic dormancy in this model. These site-specific differences in the persistence of metastatic deposits after primary tumor resection encourage future studies into this direction. Moreover, this model can be used to investigate the pathways that regulate metastatic dormancy (*e.g*., by targeting the tumor cells with small molecules or the host stroma by genetic knockout) as the DTCs mainly remain at a very low level even after surgery, but have the potential to grow out to multicellular colonies.

Based on the observation of metastatic progression of the same patient tumor during serial transplantation, we started to profile non-metastatic and metastatic C5 tumors of consecutive passages at the genome (SWGS, WES) and transcriptome (RNA-seq) level in a first set of proof-of-concept experiments. The initial results indicate that there are only very few and slight changes in the copy number variations (three loci on chromosome 5 and one locus each on chromosomes 6, 17, 18, and X). However, none of the identified chromosomal loci corresponded to any gene found to be differentially expressed by RNA-seq. Only one of two analyzed non-metastatic *vs*. metastatic pairs showed significant regulation of human mRNA at all. In contrast, both pairs harbored significant alteration of murine gene expression. These alterations must be due to changes in the murine tumor stroma, which is widely described to replace human stroma in PDX models^23,25–27^, and that might have contributed to metastatic progression during serial transplantation in our model. Of course, these first findings have to be pursued by a range of future experiments such as qPCR, WB or IHC with additional pairs of non-metastatic parental vs. metastatic filial C5 tumors. Nevertheless, they are intriguingly in line with the above-referenced, recent study by Mo *et al*. also demonstrating that metastatic *vs*. non-metastatic PCa PDX models (from different primary tumor foci of the same donor, independent of serial transplantation) cannot be discriminated by their genomic or human transcriptomic profile, but by their murine transcriptomic profile^23^. Based on the murine genes they found, the authors developed a signature of 93 stromal genes that was independently prognostic of metastasis within 5 years following prostatectomy. Of the 37 regulated murine genes we identified in the C5 model, three genes were also included in the stroma-derived metastatic signature developed by Mo *et al*. (*Des*, *Spp1*, *Pvalb*). This rather weak concordance might be explained by the different implantation sites used for PDX engraftment in the study by Mo *et al*. (subrenal capsule) and the present study (s.c.). We initially decided to apply s.c. models for two reasons: despite the rather low tumor take rates and the artificial tumor environment, s.c. xenografts have the advantage of (I) a low degree of tissue damage during a very simple, hardly invasive engraftment, which ensures that distant metastases are most likely spontaneously developed and not artificially delivered as possible during more invasive techniques (particularly since the subrenal capsule is highly vascularized compared to the subcutaneum), and (II) the opportunity to surgically resect primary tumors (which was required to investigate whether the initial DTCs stay dormant or grow out to metastatic colonies after prolonged periods).

The C5 PDX model was derived from a patient, who showed radiographic evidence of local tumor progression under anti-androgen treatment within four months after initial diagnosis. Upon prostatectomy the patient showed PSA recurrence within one month and developed lymph node and bone metastases despite continued ADT. Therefore, the tumor can be classified as castration-resistant at the time of PDX generation. As a typical feature of CRPC, the C5 tumors maintained AR expression and did not grow on female mice, which reflects that CRPC essentially remains androgen-dependent. The majority of identified mechanisms leading to CRPC involve the androgen/AR axis^28^. For instance, the remaining AR signaling in CRPC can be explained by the occurrence of AR mutations^29^, AR amplification^28^ or AR splice variants^30^, all of which are not present in the C5 tumors as assessed by SWGS, WES, qPCR, and WB. In particular, we tested AR-V7 expression in C5 tumors of different passages and detected very low levels by WB and qPCR (Suppl. Fig. S2). The only mutation found in the AR gene by WES was a silent mutation.

Moreover, it is known that even small concentrations of extra-gonadal androgens can activate the AR pathway in CRPC and that the cells can produce their own androgens^31^ which seems, however, also unlikely in the C5 PDX model since there was no growth observable in female mice. Other mechanisms of AR-dependent growth of CRPC include AR gene overexpression^32^ or increased expression of transcriptional co-activators^29^. While absolute AR expression levels were quite normal, we observed a slight increase in both p300 and the CREB-binding protein CBP in C5 tumor material (RNA-seq). We therefore speculate that the p300-CBP transcriptional co-activator complex might contribute to the castrate-resistant phenotype of the C5 tumor. Future studies will address whether therapeutic targeting of p300-CBP inhibits the C5 tumor growth as recently demonstrated for AR-driven PCa cell lines^33^. Interestingly, the patient did not respond to abiraterone treatment during the further clinical course. It is therefore likely that the C5 model resembles an abiraterone-resistant tumor or at least bears the predisposition of abiraterone resistance and might thus be a valuable tool for future research in this field. Ongoing studies, which are beyond the scope of this first description, will determine whether C5 tumor growth is actually unaltered by abiraterone therapy and whether tumor growth characteristics change in castrated male mice.

Different genomic aberrations have been determined in human PCa over the past decades (*e.g*., TMPRSS2:ERG gene fusion, PTEN-deletion, CHD1-deletion) and have resulted in the definition of different biologic subtypes^34^. The functional relevance of such aberrations still remains to be clarified in detail. In particular, there are only few human PCa models available that harbor the TMPRSS2:ERG fusion (VCaP, DuCaP^35^, HCI-H660^36^) so that the functional consequences of the androgen-regulated ERG expression can be studied in a limited number of models only. Here, the C5 model, which shows ERG overexpression, provides an important additional investigative tool for the future. For this purpose, C5 tumors were proven to grow after transient cryopreservation making the model an attractive resource for the research community. The ERG overexpression as determined by IHC in the present study correlates very closely with the presence of the TMPRSS2-ERG fusion gene as determined by FISH^37^. The TMPRSS2:ERG fusion typically coincides with PTEN deletion^34^, which is also the case in the C5 model.

One interesting future direction might be the use of PDX models to improve personalized therapies in PCa. For instance, it is meanwhile known that ERG-positive and PTEN-negative PCa cells can be radio-sensitized by Poly (ADP-ribose) polymerase (PARP) inhibition^38,39^, an observation that could be ideally investigated further in the C5 PDX model that harbors both aberrations. Another opportunity would be to make use of the robust PSMA expression in C5 primary tumors and distant lung metastases. For instance, the model could be a helpful tool for further preclinical development of PSMA targeting approaches, which has been another emerging field of research recently^40^.

Another interesting aspect worth to be mentioned is the increased metastatic spread in the less immunodeficient SCID mice compared to the more immunodeficient NSG mice. Although NK cells are functional in SCID, but not NSG mice, SCID mice developed significantly higher numbers of CTCs and almost significantly higher numbers of DTCs in the lung (Fig. 2). At other distant sites, NSG mice also did not show higher levels of DTC infiltration. This is quite surprising since NK cells display one of the major defense mechanisms against CTCs^41^. These data strongly suggest that the CTCs in the C5 model can evade the NK cell attack, which could be due to several reasons (reviewed in^41^). Furthermore, we observed relatively low tumor take rates in *pfp*^−/−^/*rag2*^−/−^ mice (although these mice lack functional B and T cells and develop perforin granzyme pathway-incompetent NK cells). This finding can be explained by the different murine backgrounds of NSG (NOD background) and *pfp*^−/−^/*rag2*^−/−^ mice (C57BL/6 background) as it is known that NOD mice bear a specific polymorphism of the signal regulatory protein-alpha (Sirpa) enabling NOD SIRPA to bind human CD47 on the tumor cells. This binding results in a ‘don’t eat me’ signaling preventing host macrophages from phagocytosing engrafted human cells. C57BL/6 SIRPA does not contain this polymorphism and therefore hardly binds human CD47^42^. This could account for increased phagocytotic activity of host macrophages against C5 tumor cells after engraftment into *pfp*^−/−^/*rag2*^−/−^ mice.

Summarized, the PCa PDX model C5 provides a novel tool to the research community as it grows on different immunodeficient backgrounds and after transient cryopreservation. The C5 model appears suitable for studies on androgen-dependent, abiraterone-resistant growth of CRPC and displays an ERG-positive, PSMA-positive, PTEN-negative PCa model. It will enable studies on metastatic dormancy in PCa and highlights the relevance of stromal factors contributing to spontaneous metastatic spread *in vivo*.

## Electronic supplementary material

Supplementary Information

human transcriptome xenograft pair 1

human transcriptome xenograft pair 2

murine transcriptome xenograft pair 1

murine transcriptome xenograft pair 2
