# Supplementary material for: Development and Characterization of a Spontaneously Metastatic Patient-Derived Xenograft Model of Human Prostate Cancer
Source: Sci Rep. 2018 Dec 3;8:17535. doi: 10.1038/s41598-018-35695-8 (PMC6277427; doi:10.1038/s41598-018-35695-8)
Supplement: Supplementary file 1 — Supplementary Information [file 41598_2018_35695_MOESM1_ESM.pdf]

Supplementary Information

Development and Characterization of a Spontaneously Metastatic Patient-Derived Xenograft Model of Human Prostate Cancer

Tobias Lange[1], Su Jung Oh-Hohenhorst[2], Simon A. Joosse[3], Klaus Pantel[3], Oliver Hahn[4], Tobias Gosau[1], Sergey A. Dyshlovoy[5,6], Jasmin Wellbrock[7], Susanne Feldhaus[1], Hanna Maar[1], Renate Gehrcke[1], Martina Kluth[8], Ronald Simon[8], Thorsten Schlomm[2,9], Hartwig Huland[2], Udo Schumacher[1]

SUPPLEMENTARY MATERIALS AND METHODS

In vitro cultivation of C5 tumor cells

Small pieces of serially transplanted tumors were minced with scalpel blades and seeded into different kinds of cell culture plastics such as 6-wells (Sarstedt, Nümbrecht, Germany), 24-wells (Greiner, Kremsmünster, Austria) or T25 cell culture flasks (Sarstedt), PCA (polycycloalkene) or glass chamber slides (Sarstedt), either uncoated or pre-treated with Matrigel® (BD) or poly-D-lysine. Some tumor samples were treated with collagenase at 37°C for 1 h prior to seeding. Several media have been tested: Dulbecco's Modified Eagle Medium (DMEM) or RPMI-1640 (both from Gibco) containing 10 or 20% fetal calf serum (FCS) and 200 IU/mL of penicillin-streptomycin (PenStrep); or TUM-Medium consisting of RPMI-1640 with Glutamax supplemented with 10% FCS, PenStrep, 0.1 mg/mL gentamycin (Biochrom AG, Berlin, Germany), 50 nmol/mL of human transferrin (Sigma-Aldrich, Steinheim, Germany), 0.01 µg/mL of bovine insulin (Sigma-Aldrich), 0.01 µg/mL of recombinant human epidermal growth factor (Peprotech, London, UK), and 0.01 µg/mL of human basic fibroblast growth factor (Peprotech) [1]; or Iscove's Modified Dulbecco's Medium (IMDM, Gibco) with Glutamax supplemented with 25 µM HEPES, 10% FCS, PenStrep, 10% horse serum and 1 µM hydrocortisone. In addition, the attempts to establish a stable cell line from C5 cells were extended by adding androgen (R1881, 100 pM, Sigma-Aldrich) alone or in combination with anti-androgen (bicalutamide, 1 µM, Sigma-Aldrich) to the culture medium.

Attachment and proliferation of tumor cells and fibroblasts was monitored by bright field microscopy. Non-adherent cell material was removed not earlier than 5 d after initial seeding by replacing the medium. If tumor cells started to proliferate, one half of the medium was replaced twice a week. For sub-cultivation, EDTA-trypsin and an enzyme-free cell dissociation buffer were tested, but some tumor cell islets were nearly non-detachable and were therefore scraped from the plastic (fibroblasts detached easier than tumor cells).

Moreover, additional samples of re-suspended C5 tumor cells were cultivated under three-dimensional growth conditions in Matrigel in the presence or absence of androgen and anti-androgen.

Protein preparation and Western blotting

Preparation of protein extracts and Western blotting were performed as described previously with slight modifications [2]. In brief, 1×10^6 of 22Rv1 cells (positive control, AR-V7-positive) or DU145 cells (negative control, AR-V7-negative) were seeded in Petri dishes (ø 6 cm, 5 mL/dish), incubated overnight and then harvested. Xenograft tumor tissues were

mechanically homogenized in liquid nitrogen. The proteins were extracted by adding lysis buffer to harvested cells or tissue homogenates (1% NP-40 [v/v], 50 mM Tris-HCl (pH 7.6), 0.88% [w/v] NaCl, 0.25% [w/v] sodium cholate, 1 mM Na_3VO_4, 0.1 mM PMSF, 1 tablet/10 mL cOmplete Mini EDTA-free EASYpacks protease inhibitors cocktail (Roche, Mannheim, Germany)). 40 µg/slot of total protein extracts of the cells and tumor samples were loaded to the precast gradient gel (4-15% Mini-PROTEAN®TGX Stain-Free™, Cat. #456-8083, Bio-Rad, Woodinville, WA, USA), subjected to electrophoresis and transferred to PVDF membrane. The membrane was consequently incubated with the primary and secondary antibody and the signal was detected as described before [2]. The antibody used: anti-AR-V7 (abcam, #198394, 1:1000), anti-β-Actin-HRP (Santa Cruz, sc-1616, 1:10000), anti-rabbit IgG-HRP (Cell Signaling, #7074, 1:5000).

AR-V7 qPCR

1 µg of total RNA was reverse transcribed using the OmniScript RT Kit (Qiagen, Venlo, Netherlands) with random nonamer primers (IBA Lifesciences, Göttingen, Germany). qRT-PCR was run on a CFX96 system (Bio-Rad, Hercules, CA, USA) with SsoAdvanced™ Universal SYBR® Green Supermix (Bio-Rad, Hercules, CA, USA) using 5 µl of cDNA per reaction. cDNA samples were quantified using the Delta Delta CT method. Prior to statistical analysis, all qRT-PCR samples were normalized to an internal reference gene (SNRPD3). The primer sequences were as follows:

ARV7 fw: TGC GCC AGC AGA AAT GAT TG
ARV7 rv: ACC TAA GCT CCT AAG CCT CA
hAR fw: AGG AAC TCG ATC CTA TCA TTG C
hAR rv: CTG CCA TCA TTT CCG GAA
SNRPD3 fw: CAG CGG ACC GAA GAG AAG AA
SNRPD3 rv: TGT TGG ACA TCT GGC AGT TCA

SUPPLEMENTARY RESULTS

In vitro cultivation of C5 tumor cells

Irrespective of the used tumor preparation protocols, cell culture plastics, pre-coatings, or media, C5 tumor cells proliferated well initially, but could not be sub-cultivated beyond passage 3 (Suppl. Fig. S1A). Likewise, viable tumor spheres with a diameter of about 100-150 µm were formed initially (within d7-d10), but did not grow out to stable, transplantable colonies (Suppl. Fig. S1B). The addition of R1881 (Metribolone, synthetic agonist of the androgen receptor), alone or in combination with bicalutamide (anti-androgen widely used in the clinic), did not improve the two- or three-dimensional *in vitro* growth of C5 colonies (Suppl. Fig. S1B+C). These treatments have been chosen in order to mimic the typical growth behavior of castrate-resistant prostate cancer cells that do not respond to ADT, but remain essentially androgen-dependent.

AR-V7 mRNA and protein expression

qPCR analyses demonstrated very weak to absent AR-V7 mRNA expression in C5 xenograft tumors (Raw Ct values ranging around 30) compared to considerable AR-FL expression in all samples. Ct values of the housekeeping gene SNRPD3 were quite similar (Suppl. Fig. S2A). Likewise, WB analyses showed very weak AR-V7 protein expression in all C5 samples compared to strong expression in the positive control, 22rv1 cells. Beta-actin was used as loading control (Suppl. Fig. S2B). The full-length blot of the cropped gel is shown in Suppl. Fig. S3.

SUPPLEMENTARY FIGURE LEGEND

Supplementary Figure S1: *In vitro* expansion of C5 PDX tumor cells. Representative images of *in vitro* culture of C5 tumor cells recovered form PDX tumors during serial transplantation. Irrespective of different enzymatic primary tumor treatments, cell culture plastics, coatings, and media (see Materials and Methods section), stable cell culture could not be established beyond d21 (A). Likewise, neither addition of androgen (R1881) alone or in combination with the anti-androgen bicalutamide (BIC) (B) nor three-dimensional growth conditions (Matrigel) (C) did result in stable growth of C5 cells *in vitro*. Note that addition of R1881 with or without BIC did not increase the sphere diameters within d9 after recovery. At later stages, spheres disintegrated comparable to the control picture from d21.

Supplementary Figure S2: AR-V7 expression in C5 PDX tumors. (A) qPCR raw Ct values of 6 different C5 tumors from varying passages for AR-V7 (splice variant 7) and AR-FL (full length) compared to SNRPD3 (housekeeping gene) gene expression. Considering a Ct value of 30 as detection limit (red dotted line), AR-V7 expression is faintly detectable in C5 tumors while AR-FL is robustly expressed (validated by IHC in Fig. 1B). In accordance, AR-V7 protein levels are detectable slightly above background level by WB in some C5 tumors compared to 22rv1 cells (positive control) and DU145 cells (negative control). Beta-actin was used as loading control (B). Different exposure times have been used for visualization of AR-V7 and beta-actin (for the corresponding full-length blot, please see Suppl. Fig. S3). Bar charts in (A) represent mean + standard deviation of n=3.

Supplementary Figure S3: Full-length blot of AR-V7 and beta-actin of protein extracts from C5 primary tumors and 22rv1 (positive control) as well as DU-145 (negative control) cells. Red arrows indicate the band of the protein of interest. The used marker (PageRuler^{TM} Plus Prestained Protein Ladder) is not supposed to produce chemiluminescence. For evaluation of the molecular weight, the fusion of the non-chemiluminescent marker picture and the chemiluminescent protein of interest picture was used. This additional information corresponds to Suppl. Fig. S2B.

SUPPLEMENTARY REFERENCES

1 Kalinina, T. *et al.* Establishment and characterization of a new human pancreatic adenocarcinoma cell line with high metastatic potential to the lung. *BMC Cancer* **10**, 295 (2010).

2 Dyshlovoy, S. A. *et al.* Proteomic profiling of germ cell cancer cells treated with aaptamine, a marine alkaloid with antiproliferative activity. *J. Proteomic Res.* **11**, 2316-2330 (2012).

Suppl. Table T1: Copy number alterations (CNA) of C5 tumors over time (p0-p7). Please see Fig. 1C also.

Chr	start	end	2014 (p0)	2015 (p3)	2016 (p7)
2	75640001	79930001	2	3	3
3	30740001	31040001	4	4	3
3	31040001	31530001	4	3	2
3	31530001	33110001	3	3	2
3	33110001	34120001	2	3	2
3	90270001	90660001	3	3	4
5	16820001	30540001	4	4	3
5	45890001	50160001	3	4	4
5	90910001	107950001	2	2	1
5	142210001	171040001	2	2	3
5	171040001	181260001	2	2	3
6	160001	12110001	3	2	2
6	12110001	19700001	3	2	3
6	19700001	21960001	3	2	3
6	21960001	39430001	3	2	3
6	40690001	44070001	3	2	3
6	44070001	61030001	3	2	2
8	30900001	38990001	3	3	2
10	80001	12130001	3	3	2
10	12130001	14470001	1	2	1
10	14470001	39550001	3	3	2
11	200001	1400001	2	1	2
14	95780001	96610001	3	4	3
15	22240001	45120001	3	2	2
15	45620001	101850001	3	2	2
16	34730001	36120001	1	2	2
17	410001	950001	2	2	2
18	130001	5850001	2	2	3
18	73040001	73840001	2	2	1
X	2790001	5160001	1	2	1
X	5160001	7260001	1	2	2
X	53650001	54640001	2	1	2
X	63810001	66220001	1	2	2

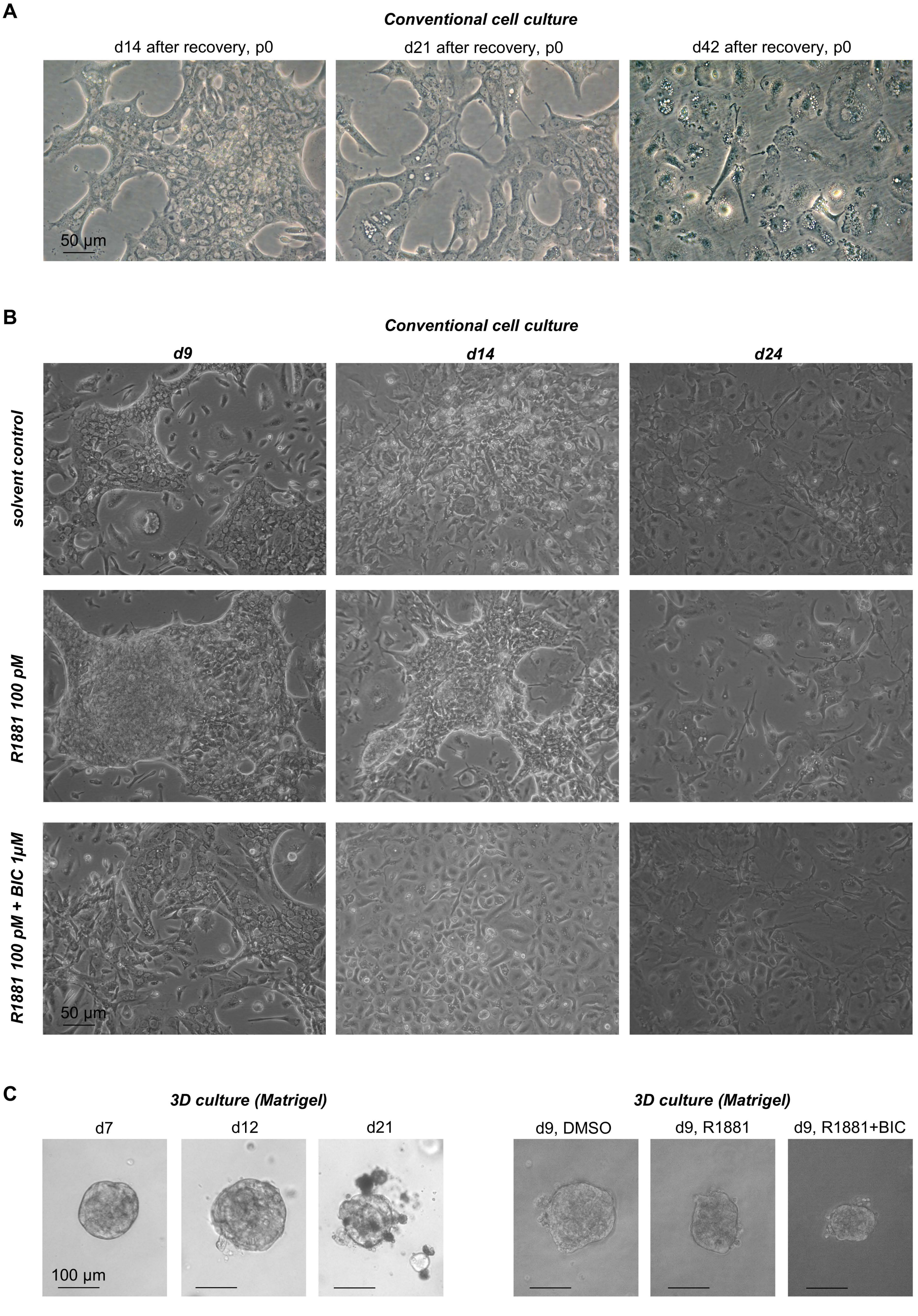
A
Conventional cell culture
d14 after recovery, p0
d21 after recovery, p0
d42 after recovery, p0
50 µm
B
Conventional cell culture
d9
d14
d24
solvent control
R1881 100 pM
R1881 100 pM + BIC 1µM
50 µm
C
3D culture (Matrigel)
d7
d12
d21
100 µm
3D culture (Matrigel)
d9, DMSO
d9, R1881
d9, R1881+BIC

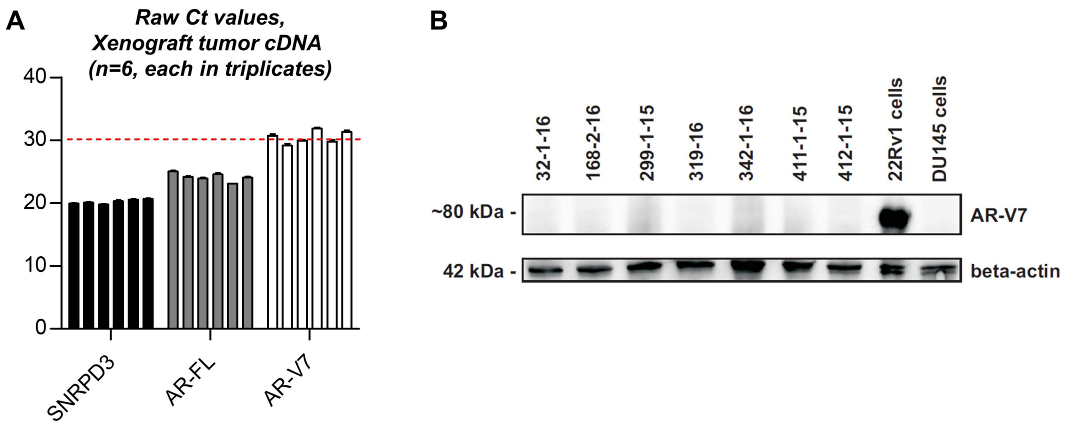

A
Raw Ct values,
Xenograft tumor cDNA
(n=6, each in triplicates)
40
30
20
10
0
SNRPD3
AR-FL
AR-V7
B
32-1-16
168-2-16
299-1-15
319-16
342-1-16
411-1-15
412-1-15
22Rv1 cells
DU145 cells
~80 kDa -
AR-V7
42 kDa -
beta-actin

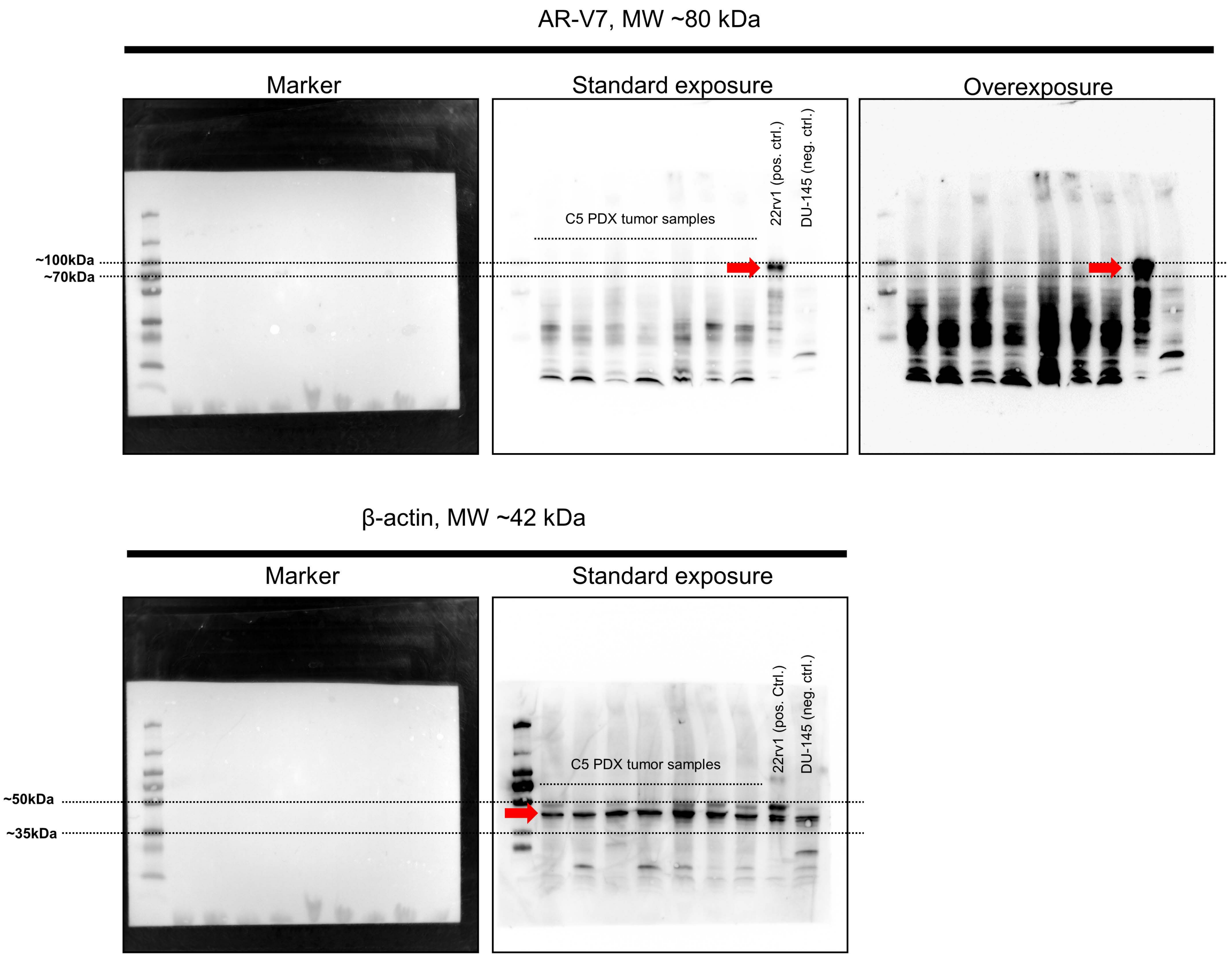
AR-V7, MW ~80 kDa
Marker
Standard exposure
Overexposure
~100kDa
~70kDa
C5 PDX tumor samples
22rv1 (pos. ctrl.)
DU-145 (neg. ctrl.)
β-actin, MW ~42 kDa
Marker
Standard exposure
~50kDa
~35kDa
C5 PDX tumor samples
22rv1 (pos. Ctrl.)
DU-145 (neg. ctrl.)
